# Proof-of-Concept of IMU-Based Detection of ICU-Relevant Agitation Motion Patterns in Healthy Volunteers

**DOI:** 10.3390/bioengineering13020164

**Published:** 2026-01-29

**Authors:** Ryuto Yokoyama, Tatsuya Hayasaka, Tomochika Harada, Si’ao Huang, Kenya Yarimizu, Michio Yokoyama, Kaneyuki Kawamae

**Affiliations:** 1 Department of Emergency and Critical Care Medicine, Yamagata University Hospital, Yamagata 990-9585, Yamagata, Japan; 2 Department of Anaesthesiology, Yamagata University Hospital, Yamagata 990-9585, Yamagata, Japan; hayasakatatsuya1101@gmail.com (T.H.); yarimizu.kenya@gmail.com (K.Y.); 3 Graduate School of Science and Engineering, Faculty of Engineering, Yamagata University, Yonezawa 992-8510, Yamagata, Japan; tharada@yz.yamagata-u.ac.jp (T.H.); tfx26958@st.yamagata-u.ac.jp (S.H.); yoko@yz.yamagata-u.ac.jp (M.Y.); 4 Ohta Nishinouchi Hospital, Koriyama 963-8558, Fukushima, Japan; kkawamae@med.id.yamagata-u.ac.jp

**Keywords:** biomechanics, wearable sensors, inertial measurement unit, agitation, intensive care unit

## Abstract

Agitation-related movements in intensive care units (ICUs), such as unintended tube removal and bed exit attempts, pose significant risks to patient safety. The wearable inertial measurement units (IMUs) offer a potential means of capturing such movements. However, the technical feasibility of discriminating ICU-relevant agitation motion patterns from multi-site IMU data remains insufficiently established. To evaluate the technical feasibility of using a convolutional neural network (CNN) applied to multi-site IMU signals to discriminate predefined ICU-relevant agitation-related motion patterns under controlled experimental conditions. Fifteen healthy volunteers performed six scripted movements designed to emulate ICU-relevant agitation-related behaviors while wearing seven IMU sensors on the limbs and waist. A CNN comprising three convolutional layers with kernel sizes of 3, 5, and 7 was trained using 1-s windows extracted from 8-s trials and evaluated using leave-one-subject-out cross-validation. The performance was summarized using macro-averaged accuracy, sensitivity, specificity, precision, and F1 score. Across 135 independent training runs, the CNN achieved a median macro-averaged accuracy of 77.0%, sensitivity of 77.0%, specificity of 95.4%, and F1 score of 77.4%. These results indicate stable window-level discrimination of the predefined motion classes under standardized conditions. This proof-of-concept study demonstrates that multi-site IMU signals combined with CNN-based modeling can technically discriminate ICU-relevant agitation-related motion patterns in a controlled laboratory setting. Although these findings do not establish clinical validity in ICU patients, they provide a methodological foundation for future studies aimed at patient-level validation and real-world critical care deployment.

## 1. Introduction

The continuous monitoring of critically ill patients in intensive care units (ICUs) is essential for ensuring patient safety. The nasogastric tubes are commonly inserted to facilitate the administration of enteral nutrition and medication [1]. However, unintended nasogastric tube removal (accidental extubation) has been reported in 35–62% of patients [2,3]. Delirium and physical restraint have been identified as risk factors for unintended nasogastric tube removal [3]. Such incidents can cause significant disadvantages among patients requiring nasogastric tubes for nutritional therapy and medication management.

In addition, patient falls occur frequently in ICUs and are reportedly associated with prolonged ICU admission and subsequent long-term hospitalizations [4]. Patients receiving intensive care are prone to agitated behavior owing to multifactorial and often overlapping causes, including delirium as well as other potentially modifiable factors such as inadequately controlled pain, sleep disruption, anxiety, respiratory failure, and patient–ventilator dyssynchrony [5,6,7]. One study reported that one-third of patients developed agitation within 7 days of ICU admission [8], whereas delirium occurred in 20–50% of ICU patients [9]. The prevention of incidents such as self-removal of nasogastric tubes and falls is of the utmost importance. Early detection of agitated behavior may serve as an effective preventive measure.

Therefore, we explored methods for identifying agitated behavior that could be applied in real-world clinical settings, focusing specifically on the use of inertial measurement unit (IMU) sensors. IMU sensors are useful for the effective assessment of human movements such as the elbow joint, cervical spine, and gait movements [10,11,12]. IMU sensors have been investigated in various medical applications, and the number of medical studies using IMU sensors has increased in recent years [13]. In particular, the utility of IMU sensors has been reported in the context of patient rehabilitation in general hospital wards [14]. Moreover, wearable accelerometer devices have shown the potential to detect differences between patients with and without delirium [15]. Such devices permit continuous and noninvasive monitoring, potentially offering various advantages such as reduced medical costs and optimized use of healthcare resources [16].

This proof-of-concept study evaluated the feasibility of using IMU sensors to detect agitation-related movements in healthy participants.

## 2. Materials and Methods

### 2.1. Study Design and Ethics

This single-center prospective study was approved by the Ethics Committee of the Faculty of Medicine, Yamagata University, on 28 April 2025 (approval Number: 2025-28, 2025-180). All participants were informed about the informed consent form processes prior to the single study visit.

### 2.2. Participant Demographics

A total of 15 healthy volunteers (13 males and 2 females; median (IQR) age, 23.0 (21.0–24.5) years) participated in this study. Four participants (26.7%) had underlying conditions such as atopic dermatitis or childhood asthma. The exclusion criteria were as follows: (1) age < 18 years; (2) history of musculoskeletal disorders or collagen diseases; (3) presence of a pacemaker; and (4) lack of consent to participate in the study. None of the participants were excluded or withdrawn during the study period.

The height, weight, and body mass index of each participant were recorded (Table 1).

### 2.3. Overview of IMU Sensors

**Figure 1:**
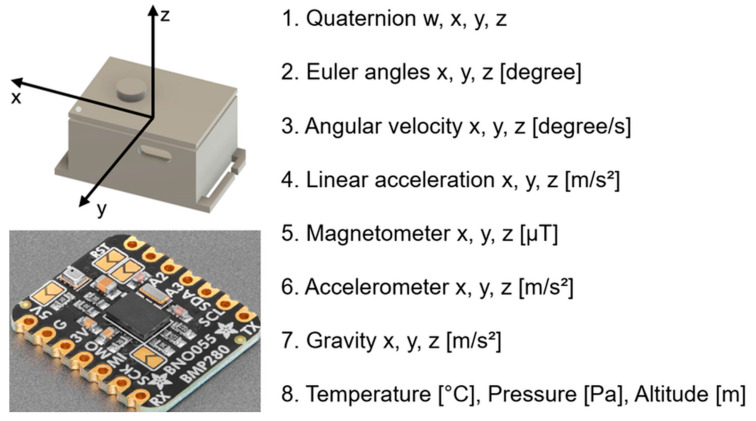
Sensor configuration and output parameters of the inertial measurement unit (IMU; BNO055 + BMP280). The IMU sensor provides multi-dimensional motion and environmental data, including quaternion, Euler angles, angular velocity, linear acceleration, magnetometer, accelerometer, gravity, temperature, pressure, and altitude. Calibration levels are represented from “0” (uncalibrated) to “3” (fully calibrated). The three-dimensional coordinate system (x, y, z) used for analysis is illustrated.

The IMU sensors used in this study were an Adafruit BNO055 Absolute Orientation Sensor (Adafruit Industries, New York, NY, USA) and a breakout board equipped with BNO055, a 9-axis IMU manufactured by Bosch; Gerhard-Kindler-Strasse 9, 72770 Reutlingen, Germany (Figure 1). Unlike conventional accelerometers, gyroscopes, and magnetometers, the BNO055 incorporates an ARM Cortex-M0 microcontroller that performs internal sensor fusion and directly outputs “Absolute Orientation” data. The device was calibrated using a proprietary algorithm developed by Bosch, the detailed mechanism of which has not been publicly disclosed.

The IMU data were collected at a sampling rate of 25 Hz using seven wearable IMU devices equipped with barometric pressure and temperature sensors (BNO055 + BMP280). As shown in Figure 1, the acceleration, angular velocity, geomagnetic field, orientation (Euler angles), quaternion, barometric pressure, temperature, and altitude (m) were captured from the ankles, waist, wrists, and upper arms during all movement tasks. Furthermore, the combined IMU system (BNO055 + BMP280) included an automatic calibration function that enabled real-time monitoring of the calibration status.

Before attaching the sensors to the participants, all sensors were calibrated according to the procedures recommended by Bosch, such as moving the sensors in a figure-eight pattern and rotating them horizontally. The calibration status of all seven IMU sensors was confirmed to be set to “3” (fully calibrated) before data collection.

### 2.4. Data Collection

**Figure 2:**
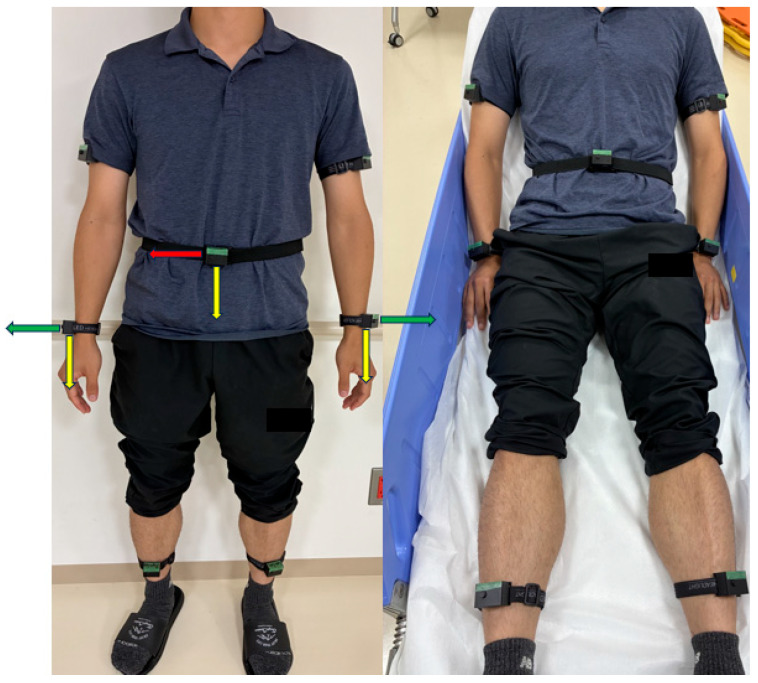
Body placement of inertial measurement unit (IMU) sensors and global axis orientation. The anatomical locations of the seven IMU sensors are schematically illustrated for standing and supine postures. The sensors were positioned at the bilateral wrists, upper arms, ankles, and waist. Detailed specifications and hardware configurations of the IMU sensors are provided in Figure 1. The x-, y-, and z-axes represent the horizontal, vertical, and depth directions, respectively, and are indicated by red, yellow, and green arrows. Limb sensors were directly affixed to the skin, whereas the waist sensor was placed over clothing. To ensure participant anonymity and neutrality, manufacturer logos were obscured.

The IMU sensors were attached to seven body locations, as follows: left and right wrists, left and right upper arms, left and right ankles, and waist (Figure 2). Each sensor recorded triaxial acceleration and triaxial angular velocity at a sampling frequency of 25 Hz for 8 s per trial. The acceleration and angular velocity components were extracted from the raw sensor signals, yielding 42 features (seven sensors × six axes).

The movements performed by the participants were defined as follows: elbow flexion (simulating unintended nasogastric tube removal), leaning forward from the bed (simulating a precursor movement to falling), and sitting up (simulating risky behaviors such as self-removal of intravenous or endotracheal tubes).

The participants performed the following six types of movements:

1. Stay: lie still on the examination bed for 10 s;
2. Remove_Tube_Right: move the right fingertip as close to the nose as possible;
3. Remove_Tube_Left: move the left fingertip as close to the nose as possible;
4. Out_Right: simultaneously extend the right arm and right leg beyond the side rail of the examination bed;
5. Out_Left simultaneously extends the left arm and leg beyond the side rail of the examination bed;
6. Sit_Up: transition from a supine to a sitting position.

**Figure 3:**
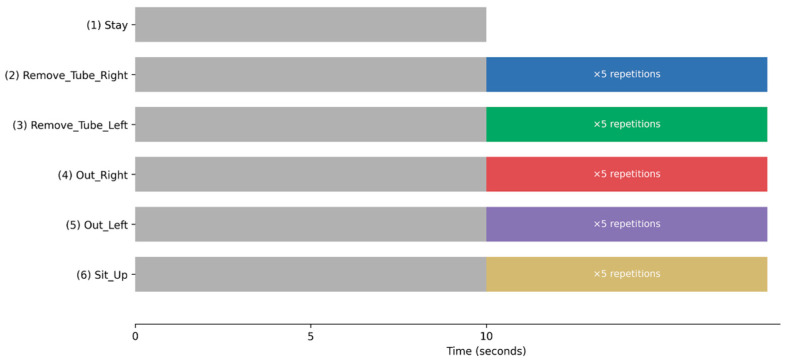
Experimental Protocol and Temporal Structure of Movement Trials. Schematic illustration of the experimental protocol and temporal structure of the movement trials. Each trial began with a mandatory 10-s static resting period (“Stay”), during which participants remained motionless on the examination bed. For all movement tasks (2–6, listed below), the resting period was followed by a predefined movement sequence that was repeated five times. A 10-s static resting period was inserted before each movement sequence to ensure clear separation between the resting and active phases and to allow consistent acquisition of baseline static behavior across trials. The movement tasks were as follows: (1) Stay; (2) Remove_Tube_Right; (3) Remove_Tube_Left; (4) Out_Right; (5) Out_Left; and (6) Sit_Up. Movement onset timing within each trial was spontaneous, allowing the capture of phase-rich temporal dynamics, including transitions between resting and active movement states. In the bar graph, Stay is represented in gray, Remove_Tube_Right in blue, Remove_Tube_Left in green, Out_Right in red, Out_Left in purple, and Sit_Up in cream.

To ensure consistency while preserving realistic movement characteristics, each experimental trial followed a standardized protocol (Figure 3). Participants were instructed to remain motionless for 10 s before initiating any movement. After this resting period, a single predefined movement was repeated five times. Importantly, a 10-s static resting period was inserted before each movement sequence, allowing clear separation between the resting and active movement phases. Although each movement was repeated five times, only an empirically determined 8-s segment containing the target movement was used for CNN input. This design ensured that the baseline static behavior was consistently captured across all trials while minimizing carryover effects between movements.

All movement tasks were demonstrated by the examiner before data collection, and the participants were instructed to imitate the demonstrated movements as naturally as possible. The trials were conducted in a predetermined order across all participants, and each participant maintained a self-consistent pace throughout the experiment.

### 2.5. Standardization of Movement Duration and Temporal Segmentation Framework

After raw IMU data were collected from all 15 participants, the actual duration of the executed movements was examined across all trials. An empirical evaluation of the raw recordings revealed that the shortest continuous movement sequence that could be reliably captured in all participants was 8 s. Based on this finding, a standardized 8-s segment was extracted from each trial, fully encompassing the intended movement.

Data-driven standardization ensured temporal consistency across participants without applying padding, trimming, or synthetic interpolation. Consequently, potential structural artifacts were avoided, and the integrity of the original sensor signals was preserved.

To further model the temporal dynamics within each movement, the standardized 8-s recordings were segmented into eight non-overlapping 1-s windows, with each window containing 25 time points. Each 1-s window was treated as an independent learning unit for model training. Consequently, a single 8-s IMU recording yielded eight discrete samples, capturing fine-grained temporal information.

### 2.6. Windowing Strategy and Phase-Rich Temporal Representation

As participants were allowed to initiate movements at spontaneous times to better reflect realistic agitation-related behavior in ICU settings, the precise onset of movement varied across trials. Consequently, individual 1-s windows within the 8-s segment can represent different phases of movement execution, including:

• Movement onset;
• Peak execution;
• Movement offset;
• Transient micro-movements;
• Static or near-static resting behavior.

This phase-rich segmentation strategy enabled the model to learn temporal progression and transitions within agitation-related movements rather than relying solely on static posture or instantaneous signal characteristics. Model exposure to heterogeneous temporal phases within each movement episode enhances dynamic motion patterns seen in real-world agitation behaviors.

All pre-processing procedures, including temporal segmentation and data structuring, were performed using Python version 3.10 (Python Software Foundation, Beaverton, OR, USA).

### 2.7. Convolutional Neural Network

#### 2.7.1. Model Structure

A convolutional neural network (CNN) was adopted for movement classification. The model input was a 25 × 42 matrix representing 25 time points and 42 features. The network consisted of three convolutional layers, each with a different kernel size (3, 5, or 7). These kernel sizes corresponded to temporal windows of approximately 0.12, 0.20, and 0.28 s, respectively. The first layer (kernel size = 3) captured short-term movements, the second layer (kernel size = 5) captured medium-range temporal patterns, and the third layer (kernel size = 7) captured longer temporal contexts. The number of filters progressively increased to 32, 64, and 128 across the layers. The final layer used a softmax activation function to classify the data into six movement categories.

#### 2.7.2. Training and Validation Protocol

A leave-one-subject-out cross-validation method was employed. In each iteration, data from one participant were used as the test set, data from three randomly selected participants were used as the validation set, and data from the remaining 11 participants were used as the training set. To account for variability in the selection of validation participants, this process was repeated nine times for each test participant, changing the combination of the three validation participants in each iteration. Consequently, 135 independent experiments (15 participants × 9 repetitions) were conducted. The model was trained for up to 100 epochs using early stopping based on the validation loss (patience = 10).

#### 2.7.3. Training Details and Reproducibility

Missing values (0.08% of all data) were handled using linear interpolation for mid-sequence gaps and forward/backward filling for sequence endpoints. All IMU features were z-score normalized. The CNN was implemented using Python 3.10 and TensorFlow/Keras 2.14.0, and trained with the Adam optimizer (learning rate = 0.001). The categorical cross-entropy loss function was used for multi-class classification. The batch size was set to 32 windows per iteration.

To reduce overfitting, batch normalization was applied after each convolutional layer, dropout regularization was used (rate = 0.3 after convolutional layers and 0.5 after the dense layer), and early stopping based on validation loss was employed with a patience of 10 epochs.

Class imbalance was not explicitly corrected using class weighting or resampling, because all six movement classes were performed the same number of times by each participant, resulting in approximately balanced class distributions.

Fixed random seeds were used for parameter initialization, data splitting, and validation subject selection to ensure reproducibility.

#### 2.7.4. Evaluation Metrics

The CNN independently classified each 1-s window. The classification performance was assessed using a confusion matrix that summarized the correspondence between the predicted labels and the ground-truth annotations. In the confusion matrix, diagonal elements indicate correctly classified samples, whereas the off-diagonal elements represent misclassifications.

The model performance was evaluated using accuracy, sensitivity, specificity, precision, and F1 score. The accuracy was calculated as the proportion of correctly classified samples among all samples. To assess overall model performance, the macro-averaged values of these metrics across the six movement classes were computed. To evaluate the detection performance for each movement class individually, each movement was treated as a positive class, whereas the remaining five movements were treated as negative classes, and the binary classification metrics were calculated accordingly.

For the final evaluation, all 135 independent experiments were sorted in ascending order of overall accuracy, and the model corresponding to the median (68th) experiment was selected as the representative model. This median-performing model lies at the 50th percentile of the full performance distribution and was therefore chosen to represent typical model behavior rather than an optimistic or pessimistic outlier. To assess the variability in model performance across all experiments, the medians and interquartile ranges (Q1–Q3) of the sensitivity, specificity, and F1 score were calculated for each movement class.

## 3. Results

### 3.1. Missing Values

Missing values accounted for 0.08% of all the data (600 of 756,000 data points). Linear interpolation was applied to fill in the missing values within the data sequence, whereas forward and backward filling were used to handle missing values at the beginning and end of the sequence.

### 3.2. Primary Outcomes (Accuracy, Sensitivity, Specificity, Precision, and F1 Score)

**Figure 4:**
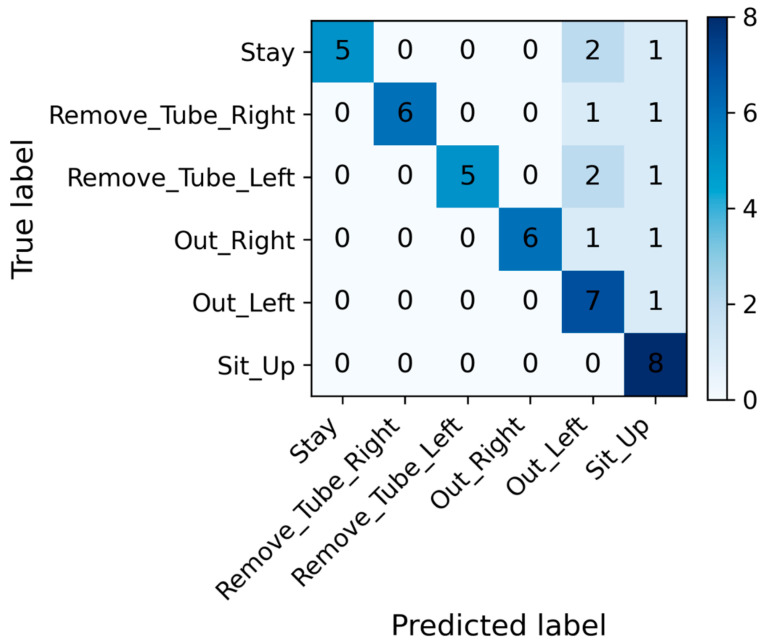
Confusion matrix of the convolutional neural network model for movement classification. The matrix shows the distribution of predicted versus true labels for each movement category. High diagonal values indicate correct classifications, whereas off-diagonal elements represent misclassifications. The confusion matrix is shown for the median-performing model (68th of 135 runs) for illustrative purposes only. The matrix is based on all 1-s windows from the test participant in that run (8 windows per movement class, total = 48 windows).

Figure 4 and Table 2 present the classification performance of the CNN model for the six movement categories. To avoid cherry-picking, 135 independent training runs were performed, and performance was summarized using median and interquartile range.

The median-performing model was used only for visualization purposes (e.g., confusion matrix) to illustrate a typical classification pattern. All quantitative conclusions in this study are based on the aggregated performance distribution across all runs, rather than on any individual model instance. The representative model, selected based on the median accuracy among 135 independent experiments, achieved a median accuracy of 77.0% (70.8–85.4%), sensitivity of 77.0% (70.8–85.4%), specificity of 95.4% (94.1–97.0%), precision of 84.5% (69.6–89.4%), and an F1 score of 77.4% (66.4–85.6%), indicating a high overall classification performance and stable discriminative capability across all movement classes. The confusion matrix indicated that Stay and Sit_Up had the highest classification accuracies, whereas Out_Right and Out_Left showed partial overlap. Overall, the model achieved high accuracy across all categories, demonstrating stable and consistent window-level discrimination of distinct movement behaviors under controlled experimental conditions. Among the six movement categories, for the Remove_Tube_Left and Remove_Tube_Right movements that simulated unintended nasogastric tube removal, specificity and precision reached a median of 100%. The median accuracy ranged from 75.0% to 87.5%, the F1 score ranged from 84.2% to 85.7%, and the sensitivity ranged from 75.0% to 87.5%. For the Out_Left and Out_Right movements, which represented bed-exiting or falling actions, the specificity and precision achieved a median of 100%, and the median F1 score ranged from 82.3% to 85.7%. The median sensitivity remained between 75.0% and 87.5%, showing a pattern similar to that of the Remove_Tube_Right/Left movements. The Sit_Up movement demonstrated high sensitivity (median 87.5%) and specificity (median 95.0%), with a precision of 80% and an F1 score of 77%. For the Stay movement, the accuracy and sensitivity reached a median of 100%, whereas the specificity had a median of 95%. However, the precision (median 61.5%) and F1 score (median 72.7%) for Stay were the lowest among all movements, indicating occasional misclassification of other movements as Stay. The full distribution of class-wise performance metrics across all 135 independent runs is provided in Supplementary Table S1.

The wide interquartile ranges for some classes (e.g., Stay) reflect variability across the 135 cross-validation runs rather than subject-level dispersion.

### 3.3. Secondary Outcome (Learning Curves)

**Figure 5:**
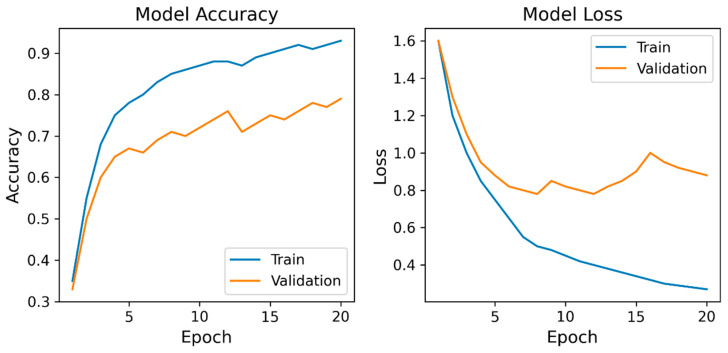
Learning curves of the convolutional neural network model. Model Accuracy: training and validation accuracy; Model Loss: training and validation loss. The curves demonstrate a steady improvement and convergence in accuracy and loss during training. Validation accuracy plateaued around 0.8, and validation loss stabilized after approximately 10 epochs, indicating stable optimization and the absence of severe overfitting.

Figure 5 shows the learning curves of the CNN model.

The training accuracy steadily increased with each epoch, reaching approximately 0.9, whereas the validation accuracy improved to approximately 0.8 before it plateaued. Correspondingly, the training and validation losses decreased markedly during the early epochs.

## 4. Discussion

The results of this proof-of-concept study indicate that attaching IMU sensors to the limbs and waist permits the feasible discrimination of predefined agitation-related movement patterns at the window level. The CNN model achieved a median macro-averaged accuracy of 77.0% (70.8–85.4%), sensitivity of 77.0% (70.8–85.4%), specificity of 95.4% (94.1–97.0%), precision of 84.5% (69.6–89.4%), and F1 score of 77.4% (66.4–85.6%), indicating that, under controlled conditions, non-agitation movements were rarely misclassified as agitation. Importantly, the reported metrics in this study should be interpreted as window-level discrimination performance rather than trial-level or patient-level agitation detection accuracy. The adjacent 1-s windows extracted from the same 8-s movement episode are temporally correlated. Hence, these samples cannot be considered statistically independent in a strict sense. Accordingly, this design may lead to optimistic performance estimates compared with a fully independent trial-level or event-level evaluation. However, the primary objective of this proof-of-concept study is not to estimate final clinical alarm accuracy, but to determine whether short-term IMU dynamics at the second scale contain sufficient information to discriminate agitation-related motion primitives. Although adjacent 1-s windows are temporally correlated, the leave-one-subject-out cross-validation ensures that no windows from the same participant appear in the training and test sets. Therefore, the reported performance reflects between-subject generalization, while temporal correlation primarily affects within-trial variance rather than cross-subject discrimination. In real ICU settings, hazardous events such as unintended tube removal or bed exit can occur within a few seconds, making second-level discrimination a clinically relevant temporal resolution. We therefore do not interpret the present results as clinical alarm performance, but rather as a feasibility assessment of whether phase-rich IMU dynamics (movement onset, peak execution, offset, and micro-movements) contain discriminative information relevant to ICU-related hazardous behaviors. These results demonstrate that multi-site IMU signals contain sufficient kinematic information to support reliable motion class separation, although they do not represent patient-level or real-world ICU performance. In an ICU alarm context, false negatives (missed hazardous movements) and false positives (unnecessary alarms) have asymmetric clinical costs. For this reason, we report not only accuracy but also sensitivity, specificity, and F1 score, and we use macro-averaged metrics to avoid dominance by the majority “Stay” class, enabling balanced evaluation across rare but clinically critical movement classes such as tube removal and bed-exit attempts. Future studies involving larger numbers of participants and ICU patients are required to enhance the robustness and future clinical validation potential of the proposed model.

For the Remove_Tube_Left and Remove_Tube_Right movements, which simulated unintended nasogastric tube removal, specificity and precision reached 100%, with accuracies ranging between 75.0% and 87.5%. Meanwhile, the F1 scores ranged between 84.2% and 85.7%. These results indicate that the model demonstrates a high level of performance in detecting critical movements. No false detections were observed during movement. False detection of agitated movements may increase the workload of the medical staff. Therefore, the absence of false detections in movements that simulate unintended nasogastric tube removal suggests that, in this controlled experimental setting, false-positive classifications for these motion classes were infrequent. However, the sensitivity ranged from 75.0% to 87.5%, indicating that not all intended movements were successfully detected. Moreover, a noticeable difference in the sensitivity and accuracy was observed between the right and left sides. This result may reflect the inherent difficulty in accurately identifying subtle upper limb movements in real clinical settings. Nevertheless, high sensitivity and specificity do not necessarily indicate that a machine learning model has superior overall accuracy [17]. Further investigations are required to determine the most appropriate model architecture for developing an agitation detection system in an ICU setting.

For the Out_Left and Out_Right movements, which simulated bed-exiting or falling actions, the specificity and precision reached a median of 100%, and the median F1 score ranged from 82.3% to 85.7%, indicating high performance. This may be because whole-body movements on the examination bed produce distinct changes in the IMU sensor signals, allowing the CNN model to clearly distinguish these actions from others. In contrast, the sensitivity remained at 75.0–87.5%, showing a pattern similar to that observed for the Remove_Tube_Right/Left movements, suggesting the possibility of missed detections. This may have been influenced by insufficient data or variability in the sensor placement, indicating that data augmentation is an important direction for future improvement.

The results for the Sit_Up movement differed from those mentioned above, showing a high sensitivity of 87.5%, which indicates that the CNN model can detect the sit-up motion with a high probability. The specificity was also high at 87.5%, indicating a low rate of false detection. However, the F1 score was relatively low at 77.7% when compared with the values for the other movements. As the F1 score is calculated as the harmonic mean of sensitivity and precision, a higher F1 score indicates that the model can accurately detect target movements with low false negatives and low false positives.

Clinically, the sit-up motion is an important early sign that often precedes bed falls, bed exits, or unintended removal of tubes and intravenous lines. Therefore, a high sensitivity rate is essential to minimize missed detections. Although the sensitivity for Sit_Up remained high in this study, such misclassification tendencies may reduce the sensitivity in more complex or noisy ICU environments, where subtle trunk movements occur more frequently. Considering the need to balance the detection performance of the device and the workload of the medical staff, appropriate threshold adjustments may also be required.

For the Stay movement, the median accuracy and sensitivity were 100%, indicating that the model reliably identified the resting state. Although specificity was high at 95.0%, the F1 score for Stay was the lowest score among all movements at 72.7%, suggesting that other movements were sometimes misclassified as “Stay”. This misclassification can be explained by short pauses between movements, which the model may have incorrectly interpreted as true resting states. From a clinical perspective, the over detection of rest is considered to carry minimal potential risk, as it is generally not expected to lead to unnecessary interventions. In practice, decision thresholds could be tuned to prioritize sensitivity for high-risk classes (e.g., Sit_Up, Out, and Remove_Tube) while tolerating a higher false-positive rate for low-risk classes such as Stay, reflecting real-world ICU alarm management.

In this model, the predicted labels tended to be biased toward the Sit_Up and Stay classes. This pattern suggests that trunk-dominant movements during other actions were sometimes misinterpreted as Sit_Up, while brief pauses or minimal motions were likely recognized as Stay. Such tendencies may reflect the intrinsic similarities in IMU signal patterns between these actions, as well as current limitations in distinguishing micromovements from true resting states. The overclassification of the Stay movement indicates the need to collect additional clinical data and include a broader range of movement patterns by increasing the number of participants. Furthermore, the misclassification of trunk-related movements as Sit_Up may reduce the ability of the system to accurately identify critical precursor behaviors, such as attempted bed exits or unintended tube removal.

To improve the overall reliability of the system, further refinements in movement label definitions, such as subdividing micromovements or introducing transitional labels, as well as optimization of the threshold settings between micromovements and rest, are required. These refinements may help reduce misclassification and enhance the robustness of the model, particularly in real-world ICU environments, where patient movements are more variable.

The learning curves of the proposed CNN model demonstrated clear and stable convergence during training. The training accuracy steadily increased with each epoch and reached approximately 0.9, whereas the validation accuracy improved to approximately 0.8 before it plateaued. Correspondingly, the training and validation losses decreased markedly during the early epochs. Overall, the learning curve patterns support the robustness of the proposed CNN architecture and indicate that the CNN learned stable discriminative features from the available IMU data without obvious signs of severe overfitting within this dataset. The gradual increase in validation accuracy and early stabilization of validation loss suggest that the model achieved an appropriate balance between fitting the training data and maintaining generalizability to other participants. These results collectively demonstrate stable learning behavior and support the validity of the proposed CNN framework.

In this study, we selected the median-performing model among the 135 independent experiments, based on overall accuracy. This representative model was chosen to avoid bias toward either the best or the worst results and to provide a fair representation of typical model performance. Although the figure presented in this study illustrates only one example, it is consistent with the central tendency of the performance distribution observed across all experiments. The median-performing model was used solely to illustrate a representative confusion pattern, whereas all quantitative conclusions are based on the distribution of performance metrics across all 135 runs.

This approach avoids cherry-picking extreme models while preserving the interpretability of typical classification behavior. Importantly, all statistical conclusions in this study are derived from the distribution of performance across all 135 independent runs, not from this representative model.

Current ICU delirium and agitation monitoring relies primarily on intermittent clinical assessments such as CAM-ICU or ICDSC, which, although validated, are subjective and cannot provide continuous or real-time detection of hazardous movements [18,19,20,21,22]. These tools are designed to diagnose delirium as a neuropsychiatric syndrome rather than to detect the specific motor behaviors that lead to adverse events such as device dislodgement or self-extubation.

More objective approaches using EEG or BIS-derived indices have been proposed for delirium monitoring, although their clinical deployment is limited by high cost, susceptibility to noise, and poor specificity for motor agitation [23,24,25,26]. Moreover, these methods primarily reflect cortical activity and do not directly quantify limb and trunk movements that cause mechanical harm.

In addition to conventional clinical scales and EEG/BIS monitoring, recent investigations have explored the use of wearable motion sensors for delirium and agitation detection. Systematic reviews suggest that wearable accelerometer devices can capture differences in activity patterns between delirious and non-delirious patients. This may help distinguish delirium subtypes through continuous monitoring of physical activity [15]. Previous research has established the feasibility of continuous actigraphy measurement in critically ill patients, demonstrating that wrist- and ankle-worn accelerometer devices can be comfortably worn and continuously record sleep and activity patterns over prolonged periods in the ICU environment [27]. In prospective studies combining actigraphy and machine learning, delirium episode detection accuracy was improved in neurologically impaired patients, suggesting that sensor-derived movement features can serve as salient behavioral biomarkers [28]. ICU-based systematic reviews of actigraphy have shown that accelerometer-derived activity levels are associated with delirium, sedation, and anxiety, supporting the potential role of wearable devices for non-invasive, continuous behavioral monitoring in critically ill populations [29]. Recent reviews on wearable devices highlight their emerging role in ICU settings for continuous, non-invasive monitoring of physiological and movement signals, which may be leveraged to detect hazardous behavioral patterns, including agitation and delirium [16].

In contrast, our approach focuses on learning high-dimensional, phase-resolved motion primitives associated with ICU-relevant hazardous behaviors using multi-site IMU signals and convolutional neural networks. Although our current data are limited to controlled simulations in healthy volunteers, this represents a methodological step beyond existing activity-based or scale-based monitoring systems by targeting the specific biomechanical signatures of adverse ICU movements rather than delirium diagnosis per se.

Importantly, this study should be interpreted as a technical feasibility demonstration rather than a validated clinical alarm system. Nonetheless, it highlights a potential new direction for ICU safety monitoring that is complementary to established delirium screening tools.

This proof-of-concept study had some limitations. First, although the proposed machine learning model demonstrated promising discrimination performance, the number of participants was limited to 15 healthy volunteers. Hence, future studies involving larger cohorts and ICU patients are required to confirm the generalizability of our findings.

Second, the movement protocol was designed to reflect realistic agitation-related behaviors in the ICU by allowing participants to initiate spontaneous movements. Although this approach enhances clinical relevance, it also introduces inter-individual variability in movement onset and duration. To ensure analytical consistency, all trials were standardized to an 8-s segment, corresponding to the minimum movement duration that could be reliably extracted based on an empirical evaluation of raw data from all participants. However, longer continuous recordings are required to evaluate the robustness of the system under prolonged real-world monitoring conditions.

Third, optical motion capture was not employed for reference motion tracking. Nevertheless, previous studies have demonstrated that IMU-based systems provide comparable accuracy for motion analysis, thereby supporting the validity of the proposed approach [30].

Fourth, agitation-related movements were defined by two researchers (R.Y. and T.H.), which may have limited the external validity of the study. However, these movements were designed to represent clinically relevant behaviors, such as unintended nasogastric tube removal and bed falls, and were further refined through interviews with experienced ICU nurses.

Fifth, although the CNN architecture demonstrated superior discrimination compared with simpler machine-learning models, its computational complexity may limit immediate real-time deployment in clinical settings. Future work should explore lightweight or hybrid architectures that preserve temporal feature extraction while reducing the computational demand.

Sixth, this study involved healthy volunteers performing controlled simulations rather than ICU patients exhibiting delirium or agitation. Accordingly, the generalizability of the system must be validated under actual clinical conditions, including variable movement patterns, sensor displacement, and environmental noise. In addition, because window-level performance exhibits variability across cross-validation runs (Supplementary Table S1), caution is required when extrapolating these results to patient-level alarm performance.

Seventh, we employed seven IMU sensors to maximize the signal diversity in this early-stage investigation. However, for practical clinical implementation, sensor reduction strategies, such as feature importance analysis and sensor selection algorithms, should be explored to improve usability and feasibility. Moreover, this study relied on the proprietary sensor-fusion outputs of the BNO055 device, which integrates accelerometer, gyroscope, and magnetometer signals using a closed-source algorithm. Although this enables convenient real-time orientation estimation, it limits interpretability because the internal fusion weights and filtering steps cannot be inspected or modified. This also affects reproducibility across different hardware platforms and firmware versions, and may constrain regulatory transparency for future clinical deployment. For these reasons, future studies should evaluate model performance using raw inertial signals and open-source sensor-fusion pipelines, which would allow full control over signal preprocessing, improve reproducibility, and facilitate regulatory and clinical validation.

Eighth, although seven IMU sensors were used to maximize signal diversity in this early-stage feasibility study, we did not perform quantitative analyses of individual sensor location or signal modality contributions. Although such analyses (e.g., sensor ablation or feature importance) are technically feasible, they were not included in the present work in order to maintain a clear focus on system-level feasibility rather than sensor optimization. For clinical implementation, reducing the number of sensors while preserving performance will be critical. Therefore, future studies should explicitly evaluate sensor-level contributions and identify minimal yet robust sensor configurations suitable for ICU use.

Additionally, the waist sensor was attached to clothing rather than directly to the skin, potentially introducing minor noise compared with limb-mounted sensors. Inter-participant variability in movement execution and occasional contact between the sensors and the side rails of the examination bed may also have contributed to additional noise.

Despite these limitations, this study provides preliminary evidence supporting the technical feasibility of IMU-based agitation detection and establishes a foundation for subsequent clinical validation and system optimization.

Based on the findings and limitations of this proof-of-concept study, we propose several directions for future research, as follows:

1. Clinical validation in ICU settings: evaluate the IMU-based system in ICU patients with delirium or agitation to assess its performance under real-world conditions, including prolonged monitoring, sensor displacement, and environmental noise;
2. Model and computational optimization: develop lightweight or hybrid architectures that maintain temporal feature extraction while enabling real-time inference on resource-constrained hardware;
3. Sensor reduction and optimization: apply sensor selection and feature importance analyses to identify the minimal sensor configurations that preserve classification performance while improving clinical feasibility; and
4. Extended and continuous monitoring analysis: investigate the robustness of the system using longer continuous recordings to capture dynamic transitions between resting and agitation-related movements.

## 5. Conclusions

The present study demonstrates the technical feasibility of discriminating predefined ICU-relevant agitation-related motion patterns from multi-site IMU data using a CNN under controlled experimental conditions. As this study does not include clinical validation in ICU patients, the findings should be interpreted as a preliminary methodological foundation for future studies aimed at patient-level validation and real-world deployment.

## Figures and Tables

**Table 1:** Characteristics of the healthy volunteers.

Characteristics	Total (N = 15)
Sex [male], n (%)	13.0 (86.7%)
Age [years], median (IQR)	23.0 (21.0–24.5)
Weight [kg], median (IQR)	66.0 (62.0–77.5)
Height [cm], median (IQR)	172.0 (165.0–176.0)
BMI [kg/m^2^], median (IQR)	23.8 (21.8–25.2)
Underlying disease, n (%) ^α^	4.0 (26.7%)

IQR: interquartile range; BMI: body mass index. α: Values are expressed as a number (percentage) unless indicated otherwise.

**Table 2:** Classification performance for each movement category.

Movement Category	Accuracy (%)	Sensitivity (%)	Specificity (%)	Precision (%)	F1 Score (%)
Overall	77.0 (70.0–85.4)	77.0 (70.8–85.4)	95.4 (94.1–97.0)	84.5 (69.6–89.4)	77.4 (66.4–85.6)
Stay	100 (0–100)	100 (0–100)	95.0 (92.5–100)	61.5 (0–77.7)	72.7 (0–84.2)
Remove_Tube_Right	75.0 (75.0–87.5)	75.0 (75.0–87.5)	100 (97.5–100)	100 (80.0–100)	85.7 (71.4–90.4)
Remove_Tube_Left	87.5 (62.5–100)	87.5 (62.5–100)	100 (95.0–100)	100 (76.3–100)	84.2 (76.9–91.1)
Out_Right	75.0 (62.5–87.5)	75.0 (62.5–87.5)	100 (97.5–100)	100 (85.7–100)	85.7 (71.4–93.3)
Out_Left	87.5 (75.0–87.5)	87.5 (75.0–87.5)	100 (95.0–100)	100 (77.7–100)	82.3 (76.5–88.8)
Sit_Up	87.5 (75.0–100)	87.5 (75.0–100)	95.0 (90.0–100)	80.0 (61.5–100)	77.7 (61.5–85.7)

Data are shown as the median (Q1–Q3).

## Data Availability

The original contributions presented in the study are included in the article, and further inquiries can be directed to the corresponding author.

## Supplementary Materials

The following supporting information can be downloaded at: https://www.mdpi.com/article/10.3390/bioengineering13020164/s1, Supplementary Table S1. Classwise performance distributions across 135 independent runs.

## Abbreviations

The following abbreviations are used in this manuscript:

ICU	Intensive care unit
IMU	Inertial measurement unit
CNN	Convolutional neural network
BMI	Body mass index
