# Supplementary material for: Proof-of-Concept of IMU-Based Detection of ICU-Relevant Agitation Motion Patterns in Healthy Volunteers
Source: Bioengineering (Basel). 2026 Jan 29;13(2):164. doi: 10.3390/bioengineering13020164 (PMC12937868; doi:10.3390/bioengineering13020164)
Supplement: Supplementary file 1 [file bioengineering-13-00164-s001.zip › bioengineering-4072835-supplementary.pdf]

Supplementary Table S1. Classwise performance distributions across 135 independent runs.

Movement class	Metric	Median	Q1	Q3
Out_Left	Accuracy (%)	87.5	75.0	87.5
	Sensitivity (%)	87.5	75.0	87.5
	Specificity (%)	100.0	95.0	100.0
	Precision (%)	100.0	77.7	100.0
	F1-score (%)	82.3	76.5	88.8
Out_Right	Accuracy (%)	75.0	62.5	87.5
	Sensitivity (%)	75.0	62.5	87.5
	Specificity (%)	100.0	97.5	100.0
	Precision (%)	100.0	85.7	100.0
	F1-score (%)	85.7	71.4	93.3
Remove_Tube_Left	Accuracy (%)	87.5	62.5	100.0
	Sensitivity (%)	87.5	62.5	100.0
	Specificity (%)	100.0	95.0	100.0
	Precision (%)	100.0	76.3	100.0
	F1-score (%)	84.2	76.9	91.1
Remove_Tube_Right	Accuracy (%)	75.0	75.0	87.5
	Sensitivity (%)	75.0	75.0	87.5
	Specificity (%)	100.0	97.5	100.0
	Precision (%)	100.0	80.0	100.0
	F1-score (%)	85.7	71.4	90.4
Sit_Up	Accuracy (%)	87.5	75.0	100.0
	Sensitivity (%)	87.5	75.0	100.0

	Specificity (%)	95.0	90.0	100.0
	Precision (%)	80.0	61.5	100.0
	F1-score (%)	77.7	61.5	85.7
Stay	Accuracy (%)	100.0	0.0	100.0
	Sensitivity (%)	100.0	0.0	100.0
	Specificity (%)	95.0	92.5	100.0
	Precision (%)	61.5	0.0	77.7
	F1-score (%)	72.7	0.0	84.2

Note: Performance is reported as median and interquartile range (Q1–Q3). All metrics were computed at the 1-s window level using leave-one-subject-out cross-validation. The percentage values are listed in table.
